# Neural Correlates of Semantic Interference and Phonological Facilitation in Picture Naming: A Systematic Review and Coordinate-Based Meta-analysis

**DOI:** 10.1007/s11065-024-09631-9

**Published:** 2024-02-06

**Authors:** Eleonora Arrigoni, Eleonora Rappo, Costanza Papagno, Leonor J. Romero Lauro, Alberto Pisoni

**Affiliations:** 1 PhD Program in Neuroscience, School of Medicine and Surgery, University of Milano-Bicocca, via Cadore 48, 29100 Monza, MB Italy; 2 Department of Psychology, University of Milano-Bicocca, P.zza dell’Ateneo Nuovo 1, 20126 Milan, MI Italy; 3 Center for Mind/Brain Sciences (CIMeC), Neurocognitive Rehabilitation Center (CeRiN), University of Trento, Via Matteo del Ben 5/b Bettini 31, 38068 Rovereto, TN Italy

**Keywords:** Picture naming, Semantic interference, Phonological facilitation, Meta-analysis, fMRI

## Abstract

Semantic interference (SI) and phonological facilitation (PF) effects occur when multiple representations are co-activated simultaneously in complex naming paradigms, manipulating the context in which word production is set. Although the behavioral consequences of these psycholinguistic effects are well-known, the involved brain structures are still controversial. This paper aims to provide a systematic review and a coordinate-based meta-analysis of the available functional neuroimaging studies investigating SI and PF in picture naming paradigms. The included studies were fMRI experiments on healthy subjects, employing paradigms in which co-activations of representations were obtained by manipulating the naming context using semantically or phonologically related items. We examined the principal methodological aspects of the included studies, emphasizing the existing commonalities and discrepancies across single investigations. We then performed an exploratory coordinate-based meta-analysis of the reported activation peaks of neural response related to SI and PF. Our results consolidated previous findings regarding the involvement of the left inferior frontal gyrus and the left middle temporal gyrus in SI and brought out the role of bilateral inferior parietal regions in PF.

**Supplementary Information:**

The online version contains supplementary material available at 10.1007/s11065-024-09631-9.

## Introduction

In the last decades, neurocognitive research on word production has made a compelling effort to unravel the complexity underlying picture naming. The main challenges of this field are dissecting all the sub-processes that lead to word production, accounting for the complex links between different processing stages, and, most importantly, understanding where, when, and how all these steps are implemented in the brain.

Indeed, the simple act of naming an object—a task frequently used to investigate word production—can be unpacked in several sub-processes, including perceptual and conceptual preparation, lemma retrieval, phonological word-form retrieval, syllabification, phonetic encoding, and articulation (Indefrey, 2011; Indefrey & Levelt, 2000, 2004; Levelt et al., 1999).

These stages have been detailed differently in various models of language production (Butterworth, 1989; Caramazza, 1997; Dell, 1986; Dell et al., 1997; Garrett, 1980; Levelt, 1989; Levelt et al., 1999). However, a common point is that picture naming complexity is influenced by several variables: besides the intrinsic aspects of the item (e.g., visual properties of the target pictures, familiarity with the object) or the psycholinguistic features (i.e., length and lexical frequency of the target object name), also the context in which picture naming occurs comes into play, making word production more or less immediate.

Experimental evidence shows that picture naming requires longer latencies when the target picture is presented together with categorically related items (i.e., belonging to the same semantic category) compared to unrelated items: this phenomenon is known as the semantic interference effect (SI) (e.g., Belke et al., 2005; Costa et al., 2005; Moss et al., 2005; Schnur & Martin, 2012; Schriefers et al., 1990). Conversely, naming latencies are shorter when naming is set in a phonologically related (i.e., when items are similar at the word-form level, sharing the same first phonemes) than in an unrelated context, the so-called phonological facilitation (PF) (e.g., Abel et al., 2009; Damian & Bowers, 2009; De Zubicaray et al., 2002; Pisoni et al., 2017; Rayner & Springer, 1986; Schriefers et al., 1990; Starreveld & La Heij, 1995, 1996).

SI and PF are inherently related to the mental lexicon organization: selection by competition models postulate that, during naming, lexical selection occurs due to spreading of activation throughout a network consisting of the concept, lemma, and word-form nodes, organized in different levels, with each concept represented as a node (Collins & Loftus, 1975; Roelofs, 1993). According to this model, the target word is retrieved by a spreading activation from stage to stage: the activation of the node representing the target concept increases, then it spreads towards the lemma level, and, finally, the lemma node with the highest level of activation is eventually selected. The same applies to word-form retrieval (Roelofs, 2018). The crucial point is that each activated node conveys a proportion of its activation to the connected nodes, thus resulting in a co-activation of representations, and the difference in the activation of the target and competitors determines the speed and accuracy at which naming occurs.

In this framework, the SI effect seems to be the result of a strong competition between the target and the co-activated nodes at the lexical level (Roelofs, 2018) or—according to alternative psycholinguistic models (e.g., Finkbeiner & Caramazza, 2006)—SI is considered dependent upon a more complex inhibition process of competitive representations occurring at the post-lexical level. On the other hand, the PF is likely to occur after lexical selection due to an over-activation of the target phonemes, boosted by the co-activation of both the target and the distractors’ word-form representations (Pisoni et al., 2017).

A potential aid in solving the debate on the functional locus of these effects may come from the analysis of their neural counterpart. However, while the behavioral aspects (i.e., the increase/decrease in naming latencies and the number of errors) of SI and PF are well-known and documented in the literature, which brain network may represent a strong candidate as the neural substrate underlying these two effects is still an open question.

To tackle this issue, neuroimaging and non-invasive brain stimulation techniques have been employed, either recording or interacting with brain activity during the performance of different picture naming paradigms, designed to selectively modulate the semantic or phonological context (Belke et al., 2005; Costa et al., 2005; Moss et al., 2005; Schnur & Martin, 2012; Schriefers et al., 1990; Vitkovitch & Cooper, 2012). In the picture-word interference (PWI) paradigm, the to-be-named picture is presented together with a written or auditory distractor word. The distractors can either be semantically or phonologically related or unrelated. The participant is asked to correctly name the figure as quickly as possible, without paying attention to the distractors (Damian & Bowers, 2009; Damian & Martin, 1999; de Zubicaray & Mcmahon, 2009; Schriefers et al., 1990; Starreveld, 2000). Instead, the blocked cyclic naming paradigm requires participants to repeatedly name a series of pictures shown in phonologically/semantically related sets, compared with mixed blocks including unrelated targets. In semantically related blocks, participants tend to increase/decrease their naming latencies according to the context in which naming occurs after the first cycle of repetitions (Belke et al., 2017). Another widely used task by studies investigating SI is the continuous naming paradigm, in which a series of pictures from different semantic categories are presented intermixed with unrelated fillers. The number of intervening trials (lag) between successive presentations of members of the same category may vary across sets. As the number of the named targets from the same category increases, the response times grow linearly (Costa et al., 2009; Howard et al., 2006; Schnur, 2014).

By employing these paradigms, fMRI (e.g., Canini et al., 2016; De Zubicaray et al., 2002; Diaz et al., 2014; Schnur et al., 2009), TMS (e.g., Krieger-Redwood & Jefferies, 2014; Sakreida et al., 2019), and tDCS (e.g. Pisoni et al., 2012, 2017) studies, together with voxel-based lesion-symptom mapping studies (e.g., Piai & Knight, 2018) have provided complementary information about the brain structures involved in the two effects.

Considering the small body of literature, there is a remarkable heterogeneity in the brain regions that may be linked to SI and PF in picture-naming tasks, thus limiting the possibility of drawing firm conclusions. Looking at the data across studies, there seems to be a lack of convergence, if not over the possible brain regions involved, at least in terms of the directionality of the effects within the neural network subtending SI and PF, i.e., whether the activity of a specific region is suppressed or enhanced at the different stages of word production. Moreover, it is still unclear whether some of the identified brain regions are more specifically related to one of the two effects, or rather, these are engaged in both, thus being possibly more related to shared processing stages in word production.

Increased activation of the left inferior frontal gyrus (LIFG) during semantically related conditions compared to unrelated ones was often reported by fMRI investigations of the SI effect (e.g., Canini et al., 2016; de Zubicaray et al., 2015), consolidating the evidence that the LIFG plays a crucial role during semantic processing, and, possibly, also during the resolution of the lexical competition due to co-activation of semantic representations. Although the LIFG seems to be prominently involved in semantic processing, it must be noted that other evidence links the activity of the LIFG to phonological processing. For example, Sakreida and coauthors (2019) found a PF reduction following the application of 5 Hz rTMS stimulation over Broca’s area. According to these findings, a functional–anatomical parcellation of the LIFG was suggested: the anterior portion of the LIFG could be involved in semantic processes, whereas the posterior part is more likely to be involved in the phonological stages (Hartwigsen et al., 2016; Klaus & Hartwigsen, 2019).

The left middle temporal gyrus (LMTG) is another possible neural substrate underlying SI: differential hemodynamic response of this region was found in several studies that compared semantically related to unrelated conditions, thus suggesting its involvement in semantic processing (e.g., De Zubicaray et al., 2001; Diaz et al., 2014; Gauvin et al., 2020; Piai et al., 2013; Rizio et al., 2017). However, it must be noted that the directionality of the BOLD signal changes is controversial across studies. For example, De Zubicaray and colleagues (2001) found increased activation of the LMTG in correspondence to the semantic condition in a PWI task, but, in a further study, de Zubicaray and McMahon (2009) found *less* activation of the same area in a different version of the same paradigm. This evidence suggests a critical role of the type of task used to tap SI and PF in the resulting pattern of brain activity.

The left superior temporal gyrus (LSTG) seems to be involved in both semantic and phonological processes. However, as in the case of the LMTG, it is unclear whether the activity of this region is suppressed or enhanced during the unfolding of the two effects. Considering its alleged role in PF, Abel and colleagues (2009) found a more robust activation related to the presentation of phonologically related distractors during a PWI task. De Zubicaray and colleagues (2002), instead, found a modulation of the BOLD signal in the opposite direction. Brain stimulation studies also support the role of the LSTG in PF: a reduction of the PF effect was reported following anodal tDCS stimulation of the LSTG, although the result was driven by an increase in naming latencies in the unrelated condition (Pisoni et al., 2017). However, this region seems to be involved in SI as well. The increase of the SI effect following anodal tDCS stimulation of this region also points to a link between LSTG activity and semantic processing (Pisoni et al., 2012). In a PWI paradigm, increased activation of the posterior LSTG was found when the to-be-named pictures were simultaneously presented with a semantically related distractor, as compared to a control, non-lexical distractor (a row of X) (De Zubicaray et al., 2001); however, Piai et al. (2013) found *less* activation of the anterior LSTG in the semantically related condition of a PWI paradigm compared to the neutral one. Again, activation changes of LSTG concerning semantic interference were reported in both directions.

Also, the inferior parietal lobule (IPL) seems to play an essential role during the processing of some specific semantic features or thematic relations of verbs (e.g., Boylan et al., 2015; de Zubicaray et al., 2013; Schwartz et al., 2011), besides being directly involved in semantic control and conflict resolution during SI (Noonan et al., 2013). Nevertheless, IPL was frequently linked to PF: several studies found a more robust activation of the area when naming occurs in a phonologically related context than with unrelated distractors (Abel et al., 2009, 2012; Diaz et al., 2014; Rizio et al., 2017). Interestingly, the phonological processing underlying PF might engage a bilateral network encompassing both left and right IPL (e.g., De Zubicaray et al., 2002).

Recently, significant contributions were made to summarize the available evidence to shed light on the SI and PF neural underpinnings, trying to account for the heterogeneity in the data. de Zubicaray and Piai (2019) reviewed the available data concerning spatial and temporal components of speech production in healthy and clinical populations. They pointed out the presence of conflicting results across investigations, finding a relatively consistent involvement of the posterior temporal lobe (MTG/STG), while the evidence about the role of LIFG was found to be less reliable and restricted to conflict resolution. Moreover, the authors pointed out some aspects that may have contributed to the inconsistencies between studies, such as the discrepancies in the experimental designs, behavioral paradigms used, methods of acquisition, and neural data analysis. Similarly, Anders et al. (2019) also observed inconsistency in the direction of neural effects, even when specifically examining studies using a version of the cyclic picture-naming paradigm for convergent results. Nozari and Pinet (2020) provided a critical review of the behavioral, neuroimaging, and electrophysiological evidence regarding the co-activation of representations in word production by summarizing the results of individual studies, often conflicting both at the behavioral and neural levels. The authors stressed the importance of using biologically plausible computational models to understand the mechanisms behind the co-activation of representations to overcome the interpretational challenges raised by the heterogeneity in the methodological choices in the paradigms used in the studies from which assumptions about competitive and non-competitive accounts of selection are derived.

Starting from this body of evidence, we took advantage of the meta-analytic approach to provide a quantitative synthesis of the available fMRI literature on SI and PF in picture naming paradigms for the first time. We aimed to identify significant anatomically coherent patterns of regional effects explicitly associated with SI and PF. Coordinate-based meta-analytic procedures are a powerful tool to synthesize and consolidate findings from individual neuroimaging studies by providing statistical measures about the degree of convergence of the data across experiments (Müller et al., 2018). Notably, coordinate-based meta-analyses are also a helpful method to explore and evaluate the results from the available fMRI studies with an unbiased eye, thus bringing out latent information that may not have been previously considered. This approach may also be helpful in providing information about the functional aspects of the investigated protocols. In particular, concerning the debate about the cognitive locus of the SI, if a post-lexical account were valid, areas linked to premotor regions (Alario et al., 2006; Tremblay & Gracco, 2009) and, to a lesser extent, the LIFG (Hocking et al., 2010) should be more consistently involved in the occurrence of the effect. These regions seem to be engaged in post-lexical selection/decision mechanisms. Conversely, one can suggest that the lexical selection account of the SI should be more linked to the activity of the middle and superior left temporal regions (de Zubicaray & McMahon, 2009; De Zubicaray et al., 2001; Hocking et al., 2009) and of the LIFG.

Although meta-analytic procedures are helpful in formally comparing data across individual studies, they are blind to some relevant methodological aspects that need to be taken into account when assessing the available evidence in the literature on a specific research topic. Therefore, in addition to our meta-analysis, we carried out a qualitative synthesis of the neuroimaging evidence investigating SI and PF. In particular, we considered three main aspects: (a) study characteristics, (b) evidence of behavioral effect related to SI and PF, and (c) methodological aspects related to neural data analysis. Then, we performed a hierarchical clustering analysis (Berlingeri et al., 2019), looking for converging patterns of activations and deactivations concerning SI and PF.

## Materials and Methods

### Data Collection

The literature review was carried out following PRISMA recommendations.

The available neuroimaging studies investigating SI and PF were identified based on a search on three databases: PubMed, Scopus, and Web of Science.

Searches were conducted entering the following queries:

- “fMRI (OR functional MR imaging OR functional magnetic resonance imaging OR functional MRI) AND semantic interference”
- “fMRI (OR functional MR imaging OR functional magnetic resonance imaging OR functional MRI) AND phonological facilitation”
- “PET (OR positron emission tomography) AND semantic interference”
- “PET (OR positron emission tomography) AND phonological facilitation”

The literature review was performed by three independent judges (EA, ER, and AP) in December 2022 and spanned a time window from 2000 to that date. The search retrieved 325 papers.

After deleting duplicates, the initial set included 95 results.

The article selection was independently and blindly run by three judges on Rayyan (Ouzzani et al., 2016), an online platform for meta-analyses and systematic reviews. Inter-rater disagreements on the eligibility of a study were solved by consensus.

The first screening was performed according to the following inclusion criteria:

- Original articles
- English text
- No single-case reports
- Neuroimaging studies (i.e., PET/fMRI)
- Studies in the language domain

At this stage, 58 articles did not meet the criteria and were excluded. The remaining 37 articles were examined based on these additional inclusion criteria:

- Studies on healthy subjects
- Studies employing naming paradigms in which co-activation of representations was obtained by manipulating the context using semantically or phonologically related items
- Studies that provided coordinates in the Montreal Neurological Institute (MNI) or Talairach (TAL) space
- Studies reporting the coordinates of a direct comparison of brain activation between the condition of interest (i.e., SI or PF) and a control condition, contrasts between the two effects, or parametric regressions (for meta-analysis only).

Nineteen papers were discarded, and three additional references were retrieved in related papers and evaluated for inclusion according to the criteria mentioned above during the study selection process.

At this stage, two out of 18 papers were not included in the meta-analysis but considered in the systematic review, as they did not fulfil the specific criteria for the quantitative synthesis.

**Fig. 1:**
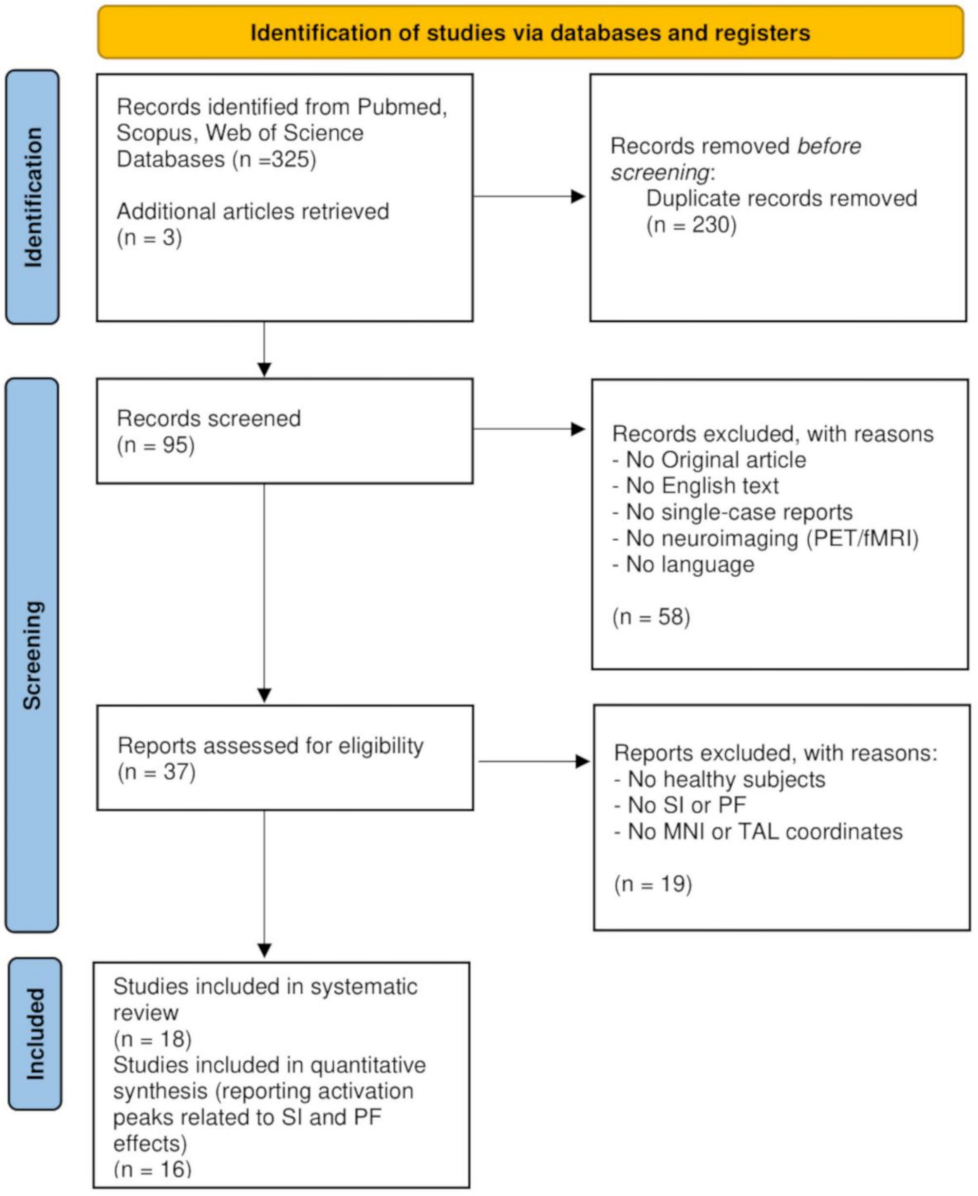
PRISMA flow chart displaying study selection steps

Ultimately, eighteen fMRI studies were considered for the systematic review, among which 16 were deemed to be eligible for the meta-analytic procedures (Fig. 1, Table 1).

**Table 1:** Summary of the studies included in the review

Study	Effect	Paradigm	Method	Sample size	Mean age (years)	Language
Abel et al. (2009), (2012)	SI/PF	PWI	fMRI (continuous imaging)	19	26; range 19–36	German
De Zubicaray et al. (2001)	SI	PWI	fMRI (sparse design)	8	28.6 (SD⫽1.8)	English
De Zubicaray et al. (2002)	PF	PWI	fMRI (sparse design)	10	26.88 (SD⫽5.04)	English
de Zubicaray and McMahon (2009)	SI/PF	PWI	fMRI (sparse design)	17	26.12 (SD//3.97)	English
de Zubicaray et al. (2013)	SI	PWI	fMRI (sparse design)	17	21.25, range = 18–27	English
Diaz et al. (2014)	SI/PF	PWI	fMRI (continuous imaging)	16	25.2, range = 19–31	English
Gauvin et al. (2020)	SI	PWI	fMRI (sparse design)	20	24, range = 18–37	English
Piai et al. (2013)	SI	PWI	fMRI (continuous imaging)	23	21.2, range = 18–29	Dutch
Rizio et al. (2017)	SI/PF	PWI	fMRI (continuous imaging)	40	Young 23.7 (4.32); elderly 67.25 (6.16)	English
Spalek and Thompson-Schill (2008)	SI	PWI	fMRI (continuous imaging)	17	21, range = 19–27	English
Canini et al. (2016)	SI	Continuous naming paradigm	fMRI (continuous imaging)	24	21.61, range = 18–26	Italian
de Zubicaray et al. (2015)	SI	Continuous naming paradigm	fMRI (perfusion imaging)	27	22.74, range = 19–34)	English
De Zubicaray et al. (2014)	SI	Blocked cyclic naming	fMRI (perfusion imaging)	25	22.93, range = 18–35	English
de Zubicaray et al. (2017)	SI	Blocked cyclic naming	fMRI (perfusion imaging)	21	20.29, range = 17–29	English
Schnur et al. (2009)	SI	Blocked cyclic naming	fMRI (sparse design)	16	Range = 18–33	English
Hocking et al. (2010)	SI	Postcue naming paradigm	fMRI (sparse design)	19	26.8, range = 20–36	English
Koester and Schiller (2011)	PF	Long-lag priming paradigm	fMRI (sparse design)	12	21.6, range = 19–29	Dutch

### Meta-analytic Procedures

By performing a meta-analysis, we aimed to identify the brain areas that were consistently activated during naming paradigms involving context manipulations typically used to elicit SI and PF effects across the selected fMRI studies. Critically, inclusion criteria did not involve the presence of positive findings at the behavioral level, not to inflate the existing evidence on the topic. For this purpose, we took advantage of a coordinate-based meta-analytic approach, namely, hierarchical clustering (Berlingeri et al., 2019), to explore clusters of converging results across studies.

Generally, the inclusion of ROI results should be avoided when conducting a coordinate-based meta-analysis and testing for spatial convergence, as ROIs inherently violate the assumption that each voxel across the whole brain has the same chance of being activated, thus leading to inflated results (Müller et al., 2018). On the other hand, for the purpose of our work, excluding ROI results may lead to biased conclusions, neglecting substantial evidence of weak but reliable effects. Indeed, considering the limited number of available studies on the topic, excluding ROI results could have led to a significant loss of information. Therefore, we conducted separate analyses, including and excluding ROI data, to compare the results and further evaluate the impact of ROI analyses in the literature concerning SI and PF effects (Gentili et al., 2019).

#### Data Extraction

From the 16 included studies, we created two separate datasets for SI and PF. For each study, all the relevant contrasts were included in the datasets (e.g., semantically related vs. unrelated, phonologically related vs. unrelated, semantically related vs. phonologically related), as well as coordinates of parametric functions of signal changes reflecting the cumulative SI effect or foci resulting from interactions between the two factors of interest. As Abel et al. (2012) re-analyzed data from Abel et al. (2009), the activation peaks of the two studies were pooled together and considered as a single study to account for the dependency of the two studies.

In total, the SI dataset included 51 foci (36 whole-brain, WB) from 10 studies (9 reported WB results) indexing increased activation and 28 foci (22 WB) from 5 studies (4 of them reporting WB results) for signal decrease. The PF dataset included 47 foci (WB data only) from 5 studies.

The coordinates in Talairach space were converted to MNI space using the Brett transform (Brett et al., 2001).

#### Hierarchical Clustering Analysis

We conducted a hierarchical clustering analysis (HC) to extract the main clusters of spatially coherent activations in relation to the SI and PF effects. This method has been recently optimized and validated by Berlingeri et al. (2019) and implemented in the Matlab toolbox “CluB—Clustering the Brain” (https://osf.io/4b2pc/).

The HC analysis considers the squared Euclidean distance between each pair of foci in the dataset and iteratively merges the clusters with minimal dissimilarity using Ward’s criterion (Ward, 1963), which minimizes intra-cluster variability after each iteration until a single cluster containing all the entered foci is obtained. This procedure generates a dendrogram, a tree-like hierarchical representation of all the clustering solutions obtained from the iterations. The algorithm then “cuts” the dendrogram at a certain level based on a spatial criterion, i.e., the maximal spatial variability considered acceptable for the purpose of the analysis, resulting in a set of clusters of activation peaks (Berlingeri et al., 2019).

We set the spatial criterion to 6 mm, as this measure is comparable to the spatial resolution of GingerALE, the most common method for activation meta-analyses (see Berlingeri et al., 2019 for a detailed discussion on CluB parameters and a formal comparison between the two meta-analytical approaches). The resulting clusters were reported specifying the *x*, *y*, and *z* coordinates of the centroid in MNI space, the standard deviation along the three axes, the cardinality (*K*, i.e., the number of foci included in each cluster), and the corresponding anatomical label, based on the Automatic Anatomic Labelling atlas. Due to the limited number of studies and, consequently, activation peaks entered in the analyses, we considered for further discussion only the clusters with a *K* ≥ 3 and at least three contributing studies to reduce the impact of single studies in the results (e.g., Devoto et al., 2018, 2020). The resulting clusters were visualized on MRIcroGL (https://www.nitrc.org/projects/mricrogl/).

## Results

### Review of fMRI Literature on SI and PF

#### Experimental Paradigm Characteristics

The majority of the selected studies (10 out of 18) investigated the neural basis of SI and PF through PWI (see Table 1). The paradigm allows examining both the SI and PF (Table Supplementary 1); among the pooled studies, 4 out of 10 considered both effects (Abel et al., 2009, 2012; de Zubicaray & Mcmahon, 2009; Diaz et al., 2014; Rizio et al., 2017).

Only two studies out of 10 (Abel et al., 2009, 2012; de Zubicaray & McMahon, 2009) employed distractors in the auditory modality, while the remaining eight used a visual (written) modality.

Considering the Stimulus Onset Asynchrony (SOA) between target and distractor, most of the studies opted for a simultaneous presentation of the two stimuli (SOA = 0 ms), thus superimposing the written word to the target picture (de Zubicaray & Mcmahon, 2009; De Zubicaray et al., 2001, 2002; Diaz et al., 2014; Gauvin et al., 2020; Piai et al., 2013; Rizio et al., 2017). Differently, de Zubicaray et al. (2013) and Abel et al. (2009, 2012) presented the distractor before the target (using SOA − 150 ms and SOA − 200 ms, respectively). Only in Spalek and Thompson-Schill (2008) the distractor was presented after the picture onset, with a late SOA (i.e., + 500 ms).

Most of the studies used unrelated items as a control condition. However, there are a few exceptions: De Zubicaray et al. (2001) compared the response to categorically related trials with a control condition in which the written distractor was a row of *X*.

In addition to the unrelated control condition, Diaz et al. (2014) included a condition in which targets and distractors had a part-whole relationship, Rizio et al. (2017) used non-words (i.e., random letter strings), and Piai et al. (2013) also included a congruent condition in which the written distractor corresponded to the name of the target.

Finally, Gauvin et al. (2020) revisited the paradigm by creating different sets in which the distractors could or could not be the target in other trials to investigate the role of LIFG in high-coactivation conditions.

Three out of 18 studies investigated SI and PF employing a blocked cyclic naming paradigm (Table S2). Schnur et al. (2009) included semantically and phonologically homogeneous blocks compared to mixed sets of stimuli. In De Zubicaray et al. (2014), SI was investigated by comparing categorically homogeneous blocks to heterogeneous/mixed blocks in which items were categorically, thematically, and phonologically unrelated. de Zubicaray et al. (2017) employed a blocked cyclic naming paradigm to examine whether homogeneous blocks of related actions elicited an SI effect similar to that observed when naming categorically related objects.

Two studies (Canini et al., 2016; de Zubicaray et al., 2015) assessed the cumulative SI using a continuous naming paradigm adapted from Howard et al. (2006) (Table S3). While de Zubicaray et al. (2015) used the original paradigm by Howard et al. (2006), Canini et al. (2016) adapted the task by presenting two different experimental lists to ensure sufficient statistical power to the er-fMRI paradigm. In the two experiments, the pictures showed items belonging to different semantic categories and a lag of *n* items randomly separated pictures from the same category.

Two additional studies were included in the review, even though the employed paradigms differed from the other considered procedures. Hocking and colleagues (2010) investigated SI at the post-lexical level by using a post-cue naming paradigm, in which two objects, semantically related or unrelated, were drawn in two different primary colors and superimposed to each other, and only after their presentation, participants were cued to name the target item based on the color of the outline.

Koester and Schiller (2011) proposed a long-lag priming paradigm in which participants were asked to name a picture after reading a prime word that was morphologically related, phonologically related, or unrelated to the target.

#### Evidence of Semantic Interference and Phonological Facilitation at the Behavioral Level

Table 2 summarizes the behavioral effects related to SI. The significant increase in naming latencies is a reliable finding across the selected studies, irrespective of the employed paradigm. Only Spalek and Thompson-Schill (2008) failed to report significant effects on RTs, possibly due to the late SOA used in their PWI paradigm (but the effect was present in the 0 ms SOA pilot experiment that was carried out outside the scanner). Canini et al. (2016) could not provide evidence of SI on RTs due to technical problems during data acquisition. SI effect on naming accuracy is rarely reported, possibly because participants often reach ceiling performances in picture naming tasks. In other words, there is no room for modulation of accuracy. Piai et al. (2013) reported lower accuracy in the semantically related condition compared to the congruent condition (i.e., targets were presented together with the corresponding written word), while they found no significant difference between semantically related and unrelated trials.

**Table 2:** Evidence of SI behavioral effects reported by each study

Study	Evidence of SI at behavioral level (RTs)	Evidence of SI at behavioral level (accuracy)	Correlation between behavioral data and neural activity
Abel et al. (2009), (2012)	Significant increase in RTs in the categorically related condition compared with unrelated, phonological, and associative distracters	Not significant	N/A
De Zubicaray et al. (2001)	Significant increase in RTs in the semantically related condition compared with lexical control (row of Xs)	N/A	N/A
de Zubicaray and McMahon (2009)	Longer naming latencies for the semantically related condition than for the other distractor types (unrelated, phonologically related)	Not significant	N/A
de Zubicaray et al. (2013)	Significant increase in naming RTs in the categorically related condition compared with unrelated distracters	N/A	N/A
Diaz et al. (2014)	Significant increase in RTs in the categorically related condition than other distracters	Not significant	Individual verbal fluency scores were positively correlated with fMRI activation to categorically related > unrelated in the right anterior STG/right IFG
Gauvin et al. (2020)	Significantly longer RTs for items paired with semantically related distracters (regardless of the response set membership condition)	N/A	N/A
Piai et al. (2013)	Slower RTs in the semantically related condition compared with the congruent and unrelated condition	Lower accuracy in the semantically related condition compared to the congruent condition. No significant difference with the unrelated condition	N/A
Rizio et al. (2017)	Slower RTs in the semantically related condition compared with the unrelated, phonologically related and nonword distracters	Not significant	N/A
Spalek and Thompson-Schill (2008)	Not significant	Not significant	N/A
Canini et al. (2016)	N/A	No evidence of CSI on error rates (lower accuracy was reported for the third repetition compared with the first and the last)	Significant correlation between accuracy scores on the fourth repetition and left caudate activity
de Zubicaray et al. (2015)	Significant increase in naming latencies with each ordinal presentation within a category, regardless of the lag	N/A	N/A
De Zubicaray et al. (2014)	Significant increase in naming latencies in categorically homogeneous compared with heterogeneous and thematically homogeneous contexts (from the 2nd presentation cycle)	N/A	N/A
de Zubicaray et al. (2017)	Significant increase in naming latencies for semantically related context compared with unrelated (from the 2nd presentation cycle)	N/A	N/A
Schnur et al. (2009)	Significant increase in naming latencies across cycles than mixed naming	N/A	Number of errors in the semantic blocked condition was positively correlated to LIFG activity. No significant relation between accuracy and activity in other ROIs (i.e., left temporal, right IFG, and ACC)
Hocking et al. (2010)	Significant increase in naming latency in the categorically related compared with unrelated condition	N/A	N/A

Interestingly, only three studies evaluated the correlation between behavioral data and neural activity, but the results are inconsistent. Canini et al. (2016) reported a significant correlation between accuracy on the fourth category repetition and left caudate activity. Schnur et al. (2009) found a positive correlation between the number of errors in the semantic-blocked condition and LIFG activity (i.e., the difference in signal between semantically blocked naming and a baseline task). In contrast, no significant relation was found between accuracy and left temporal, right inferior frontal, and anterior cingulate cortex. Diaz et al. (2014) did not directly assess the relationship between SI behavioral effects and fMRI data. However, they reported a positive correlation between individual verbal fluency scores and fMRI activation to categorically related > unrelated in the right anterior STG/right IFG.

Three of the six studies investigating PF (Table 3) did not report significant differences in naming latencies when comparing phonologically related to unrelated conditions. Crucially, Abel et al. (2009, 2012) carried out the same experiment outside the fMRI scanner and failed to observe a PF effect on RTs, thus possibly suggesting that the lack of behavioral effects was not entirely related to the fMRI setting. Instead, it is more likely that the SOA − 200 ms was too early and, consequently, non-optimal to observe PF. However, it must be noted that Rizio et al. (2017) and Diaz et al. (2014) also failed to report a significant PF effect on naming latencies using a SOA = 0 ms. Interestingly, all three studies reported differential activity at the neural level when comparing phonologically related vs. unrelated conditions. No study directly investigated the correlation between behavioral data and neural activity. However, Diaz et al. (2014) found no significant correlations between verbal fluency scores and neural activations related to the phonological condition.

**Table 3:** Evidence of PF behavioral effects reported by each study

Study	Evidence of PF at behavioral level (RTs)	Evidence of PF at behavioral level (accuracy)	Correlation between behavioral data and neural activity
Abel et al. (2009), (2012)	Not significant	Not significant	N/A
De Zubicaray et al. (2002)	Faster RTs in the orthographically/phonologically related compared with unrelated condition	N/A	N/A
de Zubicaray and McMahon (2009)	Significant decrease in RTs for the phonologically related condition compared with other distractor types (unrelated, semantically related)	Not significant	N/A
Diaz et al. (2014)	Not significantly different compared with the unrelated condition	Significantly less accurate than the unrelated trials	No significant correlations between verbal fluency scores and activation to the phonological condition
Rizio et al. (2017)	Not significantly different compared to unrelated and nonword conditions	Not significant	N/A
Koester and Schiller (2011)	Faster RTs when targets were primed by morphologically related compound words, with no difference between semantically transparent and opaque primes	N/A	N/A

#### Methodological Aspects Concerning fMRI Data Analysis

We extracted information about the type of analysis used to investigate the neural activity related to SI and PF effects, the statistical thresholding, and the number of activation peaks found at the whole-brain level. Tables S4 and S5 summarize the details of the statistical analysis carried out by each study.

Of the 18 identified studies, 16 provided adequate information about the methods adopted for multiple comparisons correction. Two studies were corrected for multiple comparisons but did not specify the correction method (De Zubicaray et al., 2001, 2002). Three studies (Koester & Schiller, 2011; Schnur et al., 2009; Spalek & Thompson-Schill, 2008) used an uncorrected *p*-*value* for statistical thresholding (Spalek and Thompson-Schill (2008) used a *p*-*value* correction using a permutation approach to localize ROIs). Ten of the included studies (Canini et al., 2016; de Zubicaray et al., 2013, 2014, 2015, 2017; Diaz et al., 2014; Gauvin et al., 2020; Hocking et al., 2010; Piai et al., 2013; Rizio et al., 2017) reported results using a cluster-level correction in conjunction to an uncorrected *p* < 0.001 or 0.005 as a cluster-forming threshold, except for Rizio et al. (2017), in which a *z* threshold of 2.3 was used: such a low primary threshold has been considered disadvantageous in terms of spatial specificity of the resulting clusters (Woo et al., 2014). Canini et al. (2016) motivated the use of a less conservative threshold (*p* < 0.005 at the voxel level, with a minimum cluster extent of *k* = 10 voxels), arguing that, when investigating semantic effects, there are higher chances of making type II errors when using a very conservative threshold.

Most of the identified studies included a WB analysis, at least as an additional exploratory analysis. In three studies, the results from the statistical comparisons between the conditions of interest were reported only at the ROI level (de Zubicaray & Mcmahon, 2009; Piai et al., 2013; Spalek & Thompson-Schill, 2008). Two studies (Diaz et al., 2014; Gauvin et al., 2020) investigating the SI effect failed to report any significant activations at the WB level by contrasting the related vs. unrelated conditions.

We acknowledged that studies investigating the neural correlates underlying SI and PF in picture naming using fMRI extensively use ROI approaches. Therefore, for all the studies that restricted the analyses to the regional level, we further reported the details of the ROI selection, specifying whether the authors made a priori selection or conducted exploratory ROI analysis, whether they clearly explained the rationale behind their ROI definition, and provided supporting literature references to substantiate their choices (Gentili et al., 2021).

Table S6 summarizes the details of the ROI selection across the studies. Sixteen out of 18 studies included in our systematic review employed ROI analysis. Thirteen studies made a priori definition of the ROI based on previous findings and clearly explained the rationale for the selection. Differently, Diaz et al. (2014) restricted the search field to eight well-established language regions only after the whole-brain exploration did not yield any significant semantic effect. Spalek and Thompson-Schill (2008) selected the ROIs based on a primary WB analysis highlighting the most active regions during experimental tasks vs. a perceptual baseline task.

In Canini et al. (2016), instead, ROIs were employed in a complementary analysis to verify whether the linear semantic increase of the LIFG and left caudate activity, resulting significant at the WB level, could also be observed at the subject level.

Three studies did not mention any information about the method used to define the ROIs (De Zubicaray et al., 2001, 2002; Schnur et al., 2009).

All the 13 studies that made a priori selections looked for differential activity patterns within the left STG/MTG. Ten out of 13 studies selected an ROI in the left IFG.

It must be noted that some studies failed to report significant effects even when restricting the analysis at the ROI level: three studies failed to report differential activity in the left IFG for the semantically related vs. unrelated comparison (De Zubicaray et al., 2014; Diaz et al., 2014; Hocking et al., 2010), while two studies failed to observe modulations of the BOLD signal after restricting the analysis to a left STG/MTG ROI (Abel et al., 2009, 2012; Koester & Schiller, 2011).

### Results: Hierarchical Clustering Analysis

#### HC: Semantic Interference ROI + WB—Signal Increase

**Fig. 2:**
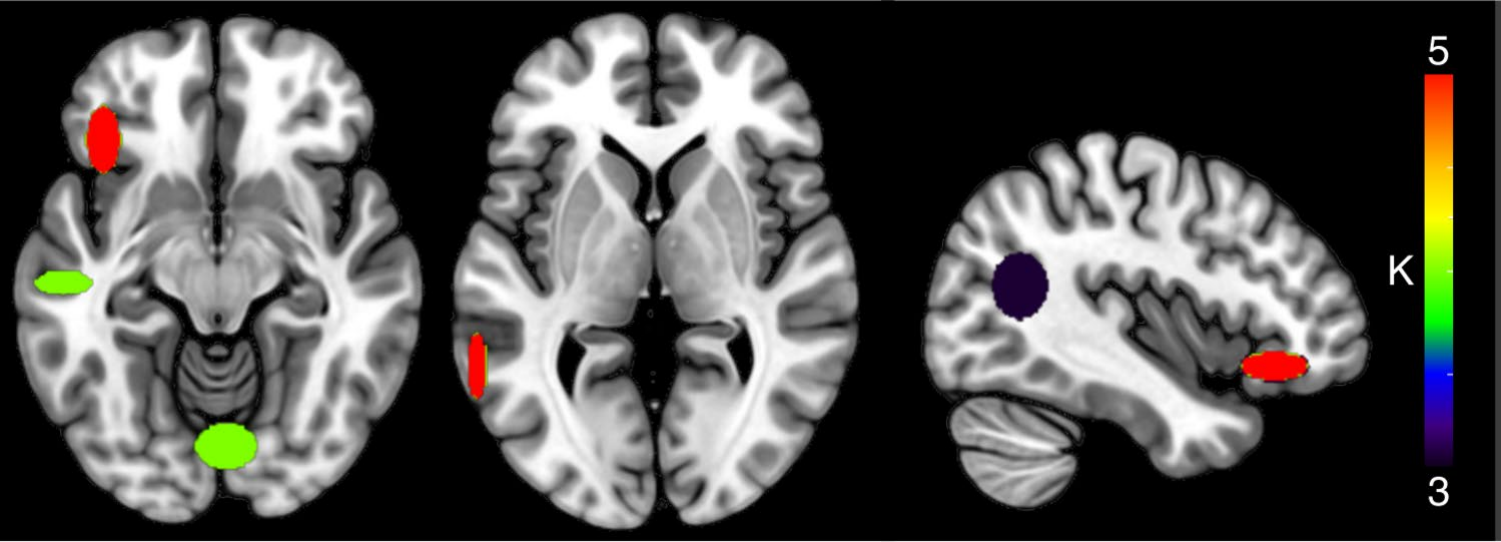
Clusters resulting from the HC analysis on SI-signal enhancement, considering both ROI and whole-brain activation foci. The color of the clusters represents cardinality values (*K*)

The cluster analysis concerning the activation peaks related to SI produced 22 clusters (five clusters with *K* ≥ 3 and at least three contributing studies, Table 4 and Fig. 2). The mean standard deviation along the three axes was 5.32 mm (*x*-axis), 5.38 mm (*y*-axis), and 4.58 mm (*z*-axis). Two clusters were found in correspondence to the LIFG pars orbitalis (five studies contributing to the cluster (Abel et al., 2012; de Zubicaray et al., 2015, 2017; Gauvin et al., 2020; Schnur et al., 2009) and the LMTG (three contributing studies (Diaz et al., 2014; Gauvin et al., 2020; Rizio et al., 2017)). Two other clusters were also found in the LMTG, both with three contributing studies each (de Zubicaray et al., 2015, 2017; De Zubicaray et al., 2001; and De Zubicaray et al., 2001; Rizio et al., 2017; Schnur et al., 2009, respectively); one cluster was found in the lingual gyrus (three contributing studies: De Zubicaray et al., 2001; Hocking et al., 2010; Rizio et al., 2017).

**Table 4:** Clusters resulting from the HC analysis on SI-signal enhancement (*K* ≥ 3 and at least three contributing studies), considering both ROI and WB activation foci

Centroid label	*X* mean	*Y* mean	*Z* mean	*X* SD (mm)	*Y* SD (mm)	*Z* SD (mm)	*K*
Left inferior frontal (pars orbitalis)	−40	29	−11	6.22	12.32	5.53	5
Left mid temporal	−62	−50	2	3.85	12.11	6.84	5
Left mid temporal	−54	−21	−11	10.68	5.01	7.50	4
Lingual	3	−78	−13	11.49	8.74	11.72	4
Left mid temporal	−41	−60	17	7.96	10.28	12.13	3

#### HC: Semantic Interference WB Only—Signal Increase

**Fig. 3:**
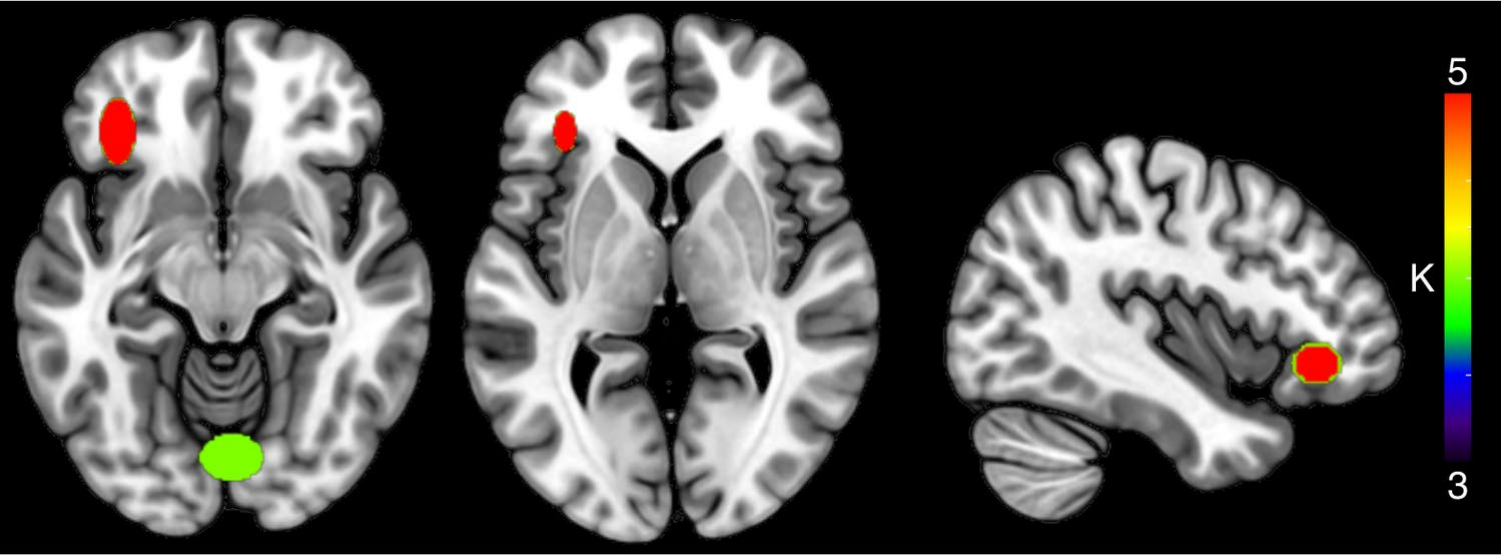
Clusters resulting from the HC analysis on SI-signal enhancement, considering only whole-brain activation foci. The color of the clusters represents cardinality values (*K*)

When considering only the activation peaks resulting from WB analyses, the HC analysis yielded 19 clusters (two clusters with *K* ≥ 3 and at least three contributing studies, Table 5 and Fig. 3). The mean standard deviation along the three axes was 6.2 mm (*x*-axis), 5.13 mm (*y*-axis), and 5.83 mm (*z*-axis). Compared to the previous solution, the LIFG pars orbitalis five contributing studies (Abel et al., 2012; Canini et al., 2016; de Zubicaray et al., 2015, 2017; Schnur et al., 2009) remained the most represented cluster, whereas the previously observed clusters in the LMTG were reduced to a single cluster (BA 21, 2 contributing studies de Zubicaray et al., 2015; Rizio et al., 2017).

**Table 5:** Clusters resulting from the HC analysis on SI-signal enhancement (*K* ≥ 3 and at least three contributing studies), considering only whole-brain activation foci

Centroid label	*X* mean	*Y* mean	*Z* mean	*X* SD (mm)	*Y* SD (mm)	*Z* SD (mm)	*K*
Left inferior frontal (pars orbitalis, BA47)	−36	33	−7	7.09	12.72	11.11	5
Lingual	3	−78	−13	11.49	8.74	11.72	4

#### HC: Semantic Interference ROI + WB—Signal Decrease

**Fig. 4:**
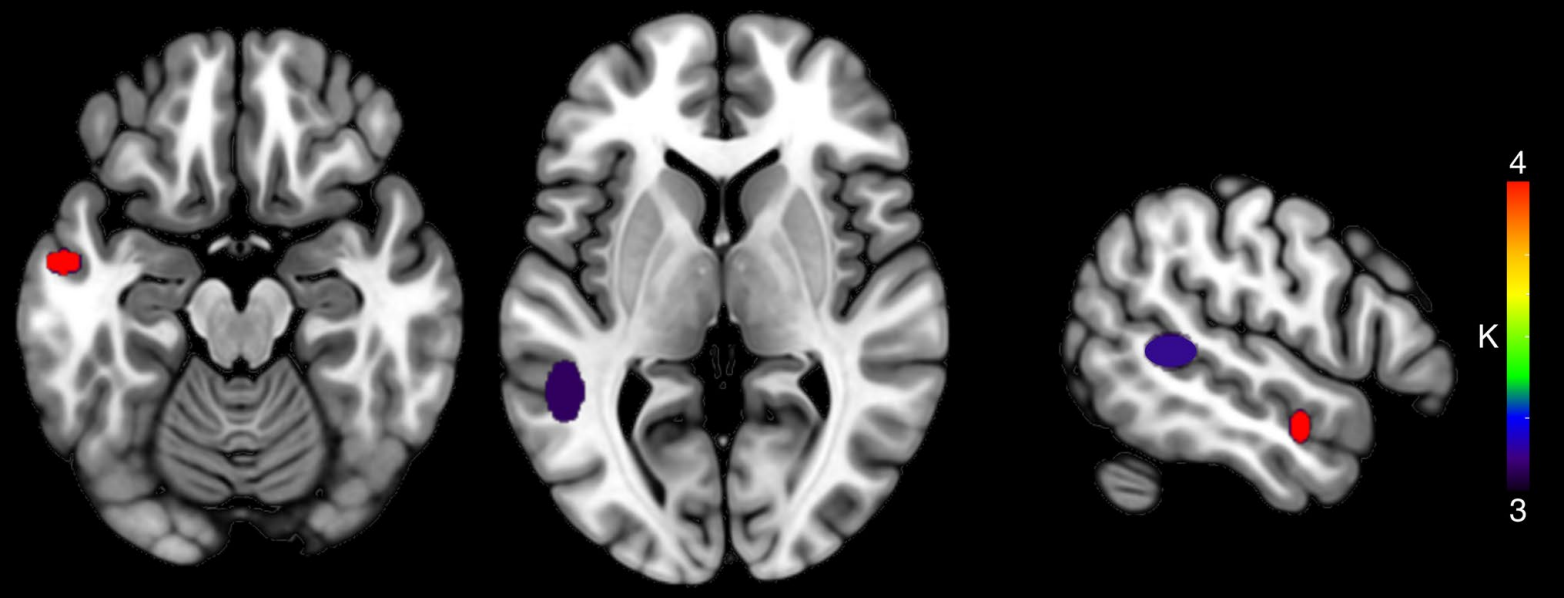
Clusters resulting from the HC analysis on SI-activation decrease, considering both ROI and whole-brain coordinates. The color of the clusters represents cardinality values (*K*)

By entering coordinates corresponding to decreased activations related to SI, we found 14 clusters (two clusters with *K* ≥ 3 and at least three contributing studies, Table 6 and Fig. 4)—mean SD along the three axes: *x* = 4.83 mm, *y* = 4.73 mm, and *z* = 5.16 mm—among which two clusters were in the LMTG (four studies contributing to cluster 1: de Zubicaray et al., 2013, 2017; De Zubicaray et al., 2014; Piai et al., 2013; three contributing studies to cluster 2: Abel et al., 2012; de Zubicaray et al., 2013, 2017).

**Table 6:** Clusters resulting from the HC analysis on SI-signal decrease (*K* ≥ 3 and at least three contributing studies), considering ROI and whole-brain coordinates

Centroid label	*X* mean	*Y* mean	*Z* mean	*X* SD (mm)	*Y* SD (mm)	*Z* SD (mm)	*K*
Left mid temporal	−56	−5	−18	5.97	0.41	6.02	4
Left mid temporal	−50	−46	6	6.60	10.66	8.23	3

#### HC: Phonological Facilitation WB Only—Signal Increase

**Fig. 5:**
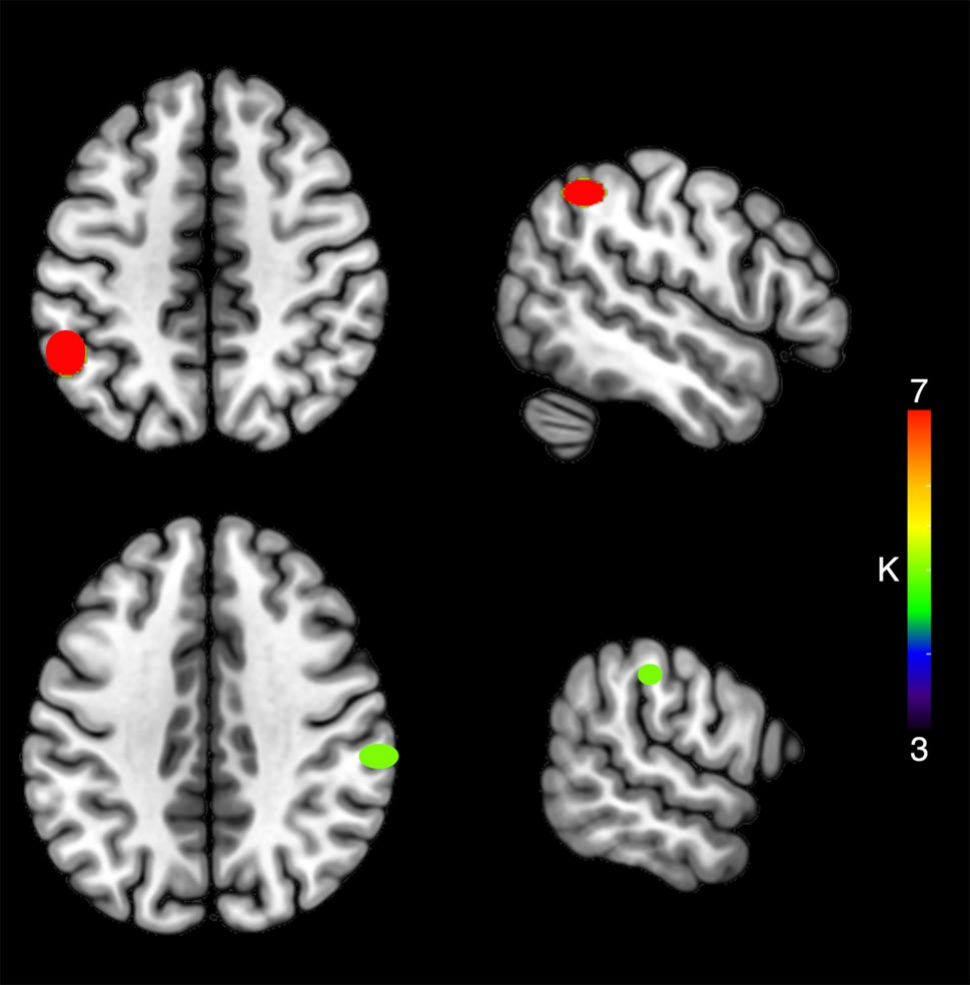
Clusters resulting from the HC analysis on PF-signal enhancement considering whole-brain only coordinates. The color of the clusters represents cardinality values (*K*)

Finally, for PF, we identified 16 clusters, with mean SD along the three axes: *x* = 3.47 mm, *y* = 3.10 mm, *z* = 3.97 mm (two clusters with *K* ≥ 3 and at least three contributing studies, Table 7 and Fig. 5) at the level of the left inferior parietal lobule (three studies, Abel et al., 2012; Diaz et al., 2014; Rizio et al., 2017) and the right supramarginal gyrus (three contributing studies; Abel et al., 2012; De Zubicaray et al., 2002; Diaz et al., 2014).

**Table 7:** Clusters resulting from the HC analysis on PF-signal enhancement (*K* ≥ 3 and at least three contributing studies), considering whole-brain coordinates

Centroid label	*X* mean	*Y* mean	*Z* mean	*X* SD (mm)	*Y* SD (mm)	*Z* SD (mm)	*K*
Left inferior parietal	−52	−51	47	7.69	8.62	5.35	7
Right supramarginal	60	−27	39	0.75	4.43	3.52	5

## Discussion

In this paper, we reviewed the current neuroimaging evidence investigating SI and PF neural underpinnings in picture naming paradigms to critically compare the methodologies employed by individual studies examining the two effects at the neural level using fMRI, searching for convergence across their findings systematically.

### SI Neural Correlates

Considering signal changes related to SI, our HC meta-analyses highlighted a convergence between studies in correspondence with the LIFG and LMTG. As outlined in the introduction, the activity of these two regions has often been reported in association with the behavioral effects related to SI. However, we found that signal enhancement of the LMTG during SI often results from a-priori-restricted ROI analyses. This finding confirms what we noticed while reviewing the ROI selection across studies: it is reasonable to believe that the effects frequently reported in SI neuroimaging studies might be too weak or too spatially dispersed, thus preventing them from reliably surviving whole-brain multiple comparisons correction.

This is not the case for LIFG, possibly due to its ubiquitous implication in top-down control and conflict resolution in the selection process of the target representation throughout the language production cascade. This result might support the role of LIFG in different aspects of the included paradigms. On the one hand, our findings support the idea that LIFG is a neural correlate of processes that are reliably engaged during response selection—including lexical selection mechanisms (Cattaneo et al., 2011; Iyer et al., 2005; Meinzer et al., 2016; Nozari & Thompson-Schill, 2013; Pisoni et al., 2012, 2015; Wirth et al., 2011). These processes might be shared throughout all the considered naming paradigms, irrespective of the differences in the experimental manipulations used to obtain the SI effect. Critically, SI-inducing protocols increase the difficulty of the lexical selection stage due to the competition between the target and the distractor, thus increasing this region's activation. Accordingly, the LMTG activity increases we found in our meta-analysis could signal a greater competition at the lexico-semantic level raised by the co-activation of semantically related representations (Diaz et al., 2014). Further investigations should clarify whether the LIFG is recruited for external, top-down control mechanisms or for internal, language-specific dynamics involved in conflict resolution, as suggested by previous studies (Pisoni et al., 2012, 2015, 2017).

On the other hand, several studies suggested a crucial role of the LIFG in semantic processing per se (e.g., Devlin et al., 2003; Thothathiri et al., 2012; Krieger-Redwood & Jefferies, 2014; Klaus & Hartwigsen, 2019; Zhu et al., 2022). Our results are also compatible with this hypothesis by showing that the LIFG may be reliably considered a cortical hub for the occurrence of a semantic effect during picture naming in semantically complex contexts.

Instead, HC on SI-related activity decrease detected converging activation patterns in the middle temporal cortex. The anterior portion of the LMTG has been related to the categorical organization of concepts in the brain (Rogers et al., 2004; Tune & Asaridou, 2016; Tyler et al., 2004). Experimental and clinical evidence suggests that lateral anterior portions of the left temporal regions may be related to semantic priming effects, i.e., signal decrease after the repetitive presentation of semantically related items (e.g., Mummery et al., 1999; Kotz et al., 2002; Rossell et al., 2003, for reviews and meta-analyses see Henson, 2003; Holderbaum et al., 2019). The decreased activation of the anterior LMTG observed in some individual studies on SI can be interpreted as a result of these priming effects, which could be a neural counterpart of the building of the SI effect occurring in this cerebral region. Conversely, a reduction in BOLD effects during SI can also be interpreted as a consequence of a lateral inhibition mechanism, which is related to internal dynamics of the spreading activation within the system: the lateral inhibition is responsible for biasing the competition towards the target representation (de Zubicaray & Mcmahon, 2009; Nozari & Pinet, 2020; Piai et al., 2013). The present results, thus, suggest that the LMTG can be involved in SI with different potential roles, i.e., as the key region where semantic priming occurs or a hub for the spread of activation cascade, where lateral inhibition may take place with increased strength during semantically related naming.

Overall, the lack of converging evidence of the involvement of motor or pre-motor regions and the clear involvement of temporal areas seems to favor the lexical selection account for the SI effect rather than a post-lexical locus. However, the variability in the included paradigms warns for more compelling evidence in this sense, potentially tailoring the behavioral protocol with the acquisition sequence to obtain robust behavioral effects in the scanner.

### PF Neural Correlates

Considering PF, we found a convergence between studies on the left IPL. This result is consistent with previous literature, as the left IPL—particularly the supramarginal gyrus—is thought to be a crucial hub for phonological processing (Church et al., 2011; Schwartz et al., 2012; Stoeckel et al., 2009).

We also identified an overlap in the right supramarginal gyrus, indicating that PF involves the activation of a bilateral parietal network. The activity of right parietal regions during the execution of tasks eliciting phonological facilitation might be enhanced by the co-activation of phonological representations, possibly reflecting a mechanism of inhibition of the non-target phonemes of the competing word forms, resulting in the target word-form being retrieved more quickly (De Zubicaray et al., 2002).

### General Considerations

Previous reviews have pointed out that the neurolinguistic literature on SI and PF lacks solid evidence about their neural correlates and their role in the extended network underlying word production (Anders et al., 2019; de Zubicaray & Piai, 2019; Nozari & Pinet, 2020). Our investigation suggests some methodological considerations that can partly help understand why a reliable pattern is not detected.

We found a striking heterogeneity across studies in terms of experimental paradigms and analyses of data. These discrepancies can remarkably impact the results and, therefore, the conclusions reached by the individual studies.

All the examined studies took advantage of a paradigm that elicits a co-activation of multiple representations leading to SI or PF; however, to achieve these effects, the experimental manipulations of the naming context widely varied across tasks, preventing the comparison of the fMRI results. It is unlikely that PWI, blocked cyclic naming, and continuous naming paradigms engage the same mechanisms. Indeed, the context manipulations proposed by the three main paradigms seem to converge at the behavioral level, increasing/decreasing naming latencies, and they possibly share most of the processing stages for word retrieval from the mental lexicon (Levelt et al., 1999). Nevertheless, each paradigm poses specific task demands variably recruiting domain-general and domain-specific mechanisms. For instance, the PWI task involves the automatic processing of distractors that can be presented in different modalities, at different timings, entailing additional processing. Instead, the blocked cyclic naming paradigm requires the continuous repetition of a small set of items, possibly being open to a strategic approach and, therefore, diverting from the typical word production cascade (Belke, 2008). This issue is of great concern from a neurolinguistic perspective, as these collateral processes certainly affect neural activity patterns observed during task performance, hence explaining the lack of consistent results. Critically, almost all studies contributing to WB LIFG cluster for signal enhancement during SI did not use a PWI paradigm but a blocked cyclic naming or continuous naming paradigm (4/5), potentially indicating that the role of this region might be related to a slow build-up of the effect, rather than a resolution of a conflict occurring at the single-trial level. Future fMRI investigations should favor naming paradigms whose design is not prominently built upon the assumptions related to a specific psycholinguistic account (be it competitive or non-competitive) to allow a better comparison and replicability of the results.

Moreover, experimental studies investigated more frequently the SI than the PF effect. The lack of attention on the neural correlates of PF could be explained by the higher agreement on the locus of this effect, namely, the word-form retrieval process, than on the SI locus, which is still controversial. This difference could have motivated the experimenters to focus more on SI neural correlates, to test the predictions based on specific accounts (de Zubicaray & Piai, 2019). However, half of the six studies that focused on brain dynamics underlying PF failed to report the expected effects on naming latencies: this limits the possibility of linking the observed activation patterns to PF. For this reason, the current fMRI evidence on PF is still too limited and, therefore, inadequate to draw any conclusion.

Another aspect that needs to be addressed is the heterogeneity of statistical procedures across studies, particularly the statistical thresholding methods used to assess significant brain activity patterns. fMRI literature on SI and PF widely relies on ROI testing. ROI analyses are extensively used in cognitive neuroscience. They increase statistical sensitivity and highlight more subtle effects that would not survive a correction for multiple comparisons at the WB level, especially in studies employing small sample sizes. Adopting an ROI approach is a valid method to test specific a-priori hypotheses. It can be helpful to disentangle differential activation patterns in complex designs (Poldrack, 2007), such as those employed in neurolinguistics fMRI literature. However, when looking for convergence across results, handling information from studies widely relying on the ROI approach might be problematic (Gentili et al., 2019). The heterogeneity of the methods used for restricting the search coverage across studies poses some limits in terms of the generalization of the results.

The overrepresentation of some specific regions across studies investigating the neural activity related to a particular effect may lead to underestimating the relevance of other, less expected, activation patterns possibly resulting from WB analysis (Gentili et al., 2019).

In addition, it must be noted that, when correcting for multiple comparisons at the WB level, part of the included experiments failed to report significant effects within regions of great interest for the investigators, which are frequently a priori assumed to be involved in SI and PF (e.g., LIFG and LMTG). This does not necessarily mean that the activity of these regions is unaffected by the co-activation of representations elicited by complex naming paradigms. Still, it suggests that the changes in BOLD signal related to SI and PF might be subtle and require sufficient statistical power to be reliably detected.

Finally, our review underscores the need for new research in this area, considering the methodological aspects highlighted in this paper. Our results obtained at the meta-analytic level, utilizing a coordinate-based approach, are challenging to generalize given the low number of studies included and should be considered exploratory. An intriguing alternative is the image-based approach, leveraging hierarchical mixed effects models to better address intra-study and random inter-study variance (Salimi-Khorshidi et al., 2009). However, this method requires whole-brain statistical images, typically unavailable unless shared by authors on open repositories (e.g., https://neurovault.org/). Unfortunately, in this case, the absence of data prevented us from using this approach. In the future, it would be valuable to revisit studies implementing meta-analytical image-based strategies, enabling more robust conclusions to be derived.

## Conclusions

The current fMRI evidence concerning the neural correlates of SI and PF is inconclusive. In this paper, we provided an overview of the available neuroimaging data, considering the existing methodological limitations that hamper the identification of solid and reproducible patterns of effects, namely, the heterogeneity of behavioral paradigms used by individual investigations, the preferential use of ROI restrictions during neural data analysis, and the inconsistency of the reported behavioral effects. Albeit exploratory, our meta-analytic synthesis supports the relative convergence among studies in correspondence with the LIFG and LMTG in relation to the SI effect, and it brings out the involvement of the bilateral IPL in the PF effect. The present results thus suggest that the LMTG can be involved in SI with different potential roles, i.e., as the key region where semantic priming occurs or a hub for the spread of activation cascade, where lateral inhibition may take place with an increased strength during semantically related naming. Future studies are necessary to clarify the role of these regions in the cascade of processes occurring during picture naming, to better inform more comprehensive neurocognitive models of word production.

## Supplementary Information

Below is the link to the electronic supplementary material.

Supplementary file1 (DOCX 30 KB)

## Data Availability

The datasets generated during and analyzed during the current study are available from the corresponding author on reasonable request.
