# Supplementary material for: Neural Correlates of Semantic Interference and Phonological Facilitation in Picture Naming: A Systematic Review and Coordinate-Based Meta-analysis
Source: Neuropsychol Rev. 2024 Feb 6;35(1):35–53. doi: 10.1007/s11065-024-09631-9 (PMC11965239; doi:10.1007/s11065-024-09631-9)
Supplement: Supplementary file 1 — Supplementary file1 (DOCX 30 KB) [file 11065_2024_9631_MOESM1_ESM.docx]

*Supplementary Information for:*

**Neural correlates of semantic interference and phonological facilitation in picture naming: a systematic review and coordinate-based meta-analysis.**

Eleonora Arrigoni^1^, Eleonora Rappo^2^, Costanza Papagno^3^, Leonor J Romero Lauro^2^, Alberto Pisoni^2†^

^1^Department of Medicine and Surgery, University of Milano-Bicocca, via Cadore 48, 29100, Monza, MB

^2^Department of Psychology, University of Milano-Bicocca, P.zza dell’Ateneo Nuovo 1, 20126, Milano, MI

^3^Center for Mind/Brain Sciences (CIMeC), University of Trento, Neurocognitive Rehabilitation Center (CeRiN),Via Matteo del Ben 5/b Bettini 31, 38068, Rovereto, TN

*Table S1: Summary of the* *characteristics of the picture-word-interference paradigms used in the selected studies.*

| Study | Effect | SOA | Distractor type | Control condition |
| --- | --- | --- | --- | --- |
| *Abel et al., 2009,2012* | SI/PF | -200 ms | Auditory | Unrelated |
| *De Zubicaray et al., 2001* | SI | 0 ms | Written | Row of Xs |
| *De Zubicaray et al., 2002* | PF | 0 ms | Written | Unrelated |
| *De Zubicaray & McMahon, 2009* | SI/PF | 0 ms | Auditory | Unrelated |
| *De Zubicaray et al., 2013* | SI | -150 ms | Written | Unrelated |
| *Diaz et al., 2014* | SI/PF | 0 ms | Written | Unrelated |
| *Gauvin et al., 2020* | SI | 0 ms | Written | Unrelated |
| *Piai et al., 2013* | SI | 0 ms | Written | Unrelated; Congruent |
| *Rizio et al., 2017* | SI/PF | 0 ms | Written | Unrelated; Non-words |
| *Spalek & Thompson-Schill, 2008* | SI | +500 ms | Written | Unrelated |

*Table S2: Summary of the* *characteristics of the blocked cyclic naming paradigms used in the selected studies.*

| Study | Effect | Control condition | Number of cycles |
| --- | --- | --- | --- |
| *De Zubicaray et al., 2014* | SI | Heterogeneous blocks | 6 |
| *De Zubicaray et al., 2017* | SI | Heterogeneous blocks | 6 |
| *Schnur et al., 2009* | SI/PF | Heterogeneous blocks | 4 |

*Table S3: Summary of the* *characteristics of the continuous naming paradigms used in the selected studies.*

| Study | Effect | Targets/fillers | # Lags |
| --- | --- | --- | --- |
| *Canini et al., 2016* | SI | 120 (5x24 categories) / 45 | 2-4-6-8 |
| *De Zubicaray et al., 2015* | SI | 120 (5x24 categories) / 45 | 2-4-6-8 |

*Table S4: Details of the fMRI analyses and summary of the results indicating signal enhancements. *= signal increase found at ROI level*

| **Study** | **Paradigm** | **Effect**  **(SI/PF)** | **Type of analysis** | **Statistical thresholding** | **Regions in which increased activity was reported** | **ROI analysis** |
| --- | --- | --- | --- | --- | --- | --- |
| *Abel et al., 2009, 2012* | PWI | SI | Semantically related>unrelated;  Semantically related>phonologically related | P<0.05 FDR corrected  (Abel 2009: ROI analysis: uncorrected p<0.001) | left inferior/middle frontal gyrus | In Abel et al. 2009: ROIs  selected from a predefined atlas |
|  |  | PF | Phonologically related>unrelated;  Phonologically related>semantically related |  | Left IPL, bilateral MTG, left middle frontal gyrus, left precuneus |  |
| *De Zubicaray et al., 2001* | PWI | SI | Semantically related> control (row of Xs) | P<0.05 corrected (not specified) | Left* and right MTG, left STG*, right medial frontal gyrus*, left ACC*, left* and right superior frontal gyrus, left IPL, right occipital lobe | VOI correction based on a priori hypotheses |
| *De Zubicaray et al., 2002* | PWI | PF | Ortographically related>unrelated | P<0.05 corrected (not specified) | Right ACC, right middle and superior frontal gyrus, right postcentral gyrus, IPL, and occipital lobe. | VOI correction based on a priori hypotheses |
| *De Zubicaray and McMahon, 2009* | PWI | SI |  | P<0.05 FDR corrected | Left middle occipital gyrus and cuneus | SVC on predefined ROIs based on a priori hypotheses |
|  |  | PF |  |  | left middle occipital gyrus and cuneus |  |
| *Diaz et al., 2014* | PWI | SI | Semantically related>phonologically related | Cluster forming threshold p<0.001;  P<0.05 corrected at cluster-level (GRF) | Left posterior MTG* | ROI analysis in eight well-established language regions |
|  |  | PF | Phonologically related>unrelated;  Phonologically related>semantically related |  | Bilateral supramarginal gyrus, angular gyrus, and left lateral occipital cortex.  left insula left central operculum and planum temporale, and right occipital fusiform gyrus. |  |
| *Gauvin et al., 2020* | PWI | SI | Semantically related>unrelated | Cluster forming threshold p<0.001;  P<0.05 FWE cluster-level | left posterior middle and superior temporal gyri*, left IFG pars orbitalis*. | Confirmatory SVC based on previous fMRI studies |
| *Rizio et al., 2017* | PWI | SI | Semantically related>unrelated;  Semantically related>phonologically related | Cluster forming threshold z>2.3;  P<0.05 corrected at cluster-level GRF | Bilateral middle frontal gyrus, left precentral, bilateral MTG, middle precuneus, right angular gyrus, bilateral occipital cortex, left cerebellum. |  |
|  |  | PF | Phonologically related>unrelated;  Phonologically related>semantically related |  | bilateral angular and supramarginal gyri, right lateral occipital cortex, left superior parietal lobule, right heschl’s, putamen, insula and central opercular cortex. |  |
| *Spalek & Thompson-Schill, 2008* | PWI | SI |  | P<0.05 uncorrected | Right posterior cerebellum*, left fusiform/parahippocampal gyri*. | ROI defined based on **WB** analysis (regions more active during the experimental task compared to baseline) |
| *Canini et al., 2016* | Continuous naming paradigm | SI | Linear increase in BOLD response for the category repetition modulator | P<0.005 voxel level (minimum k=10) | left IFG and caudate | Complementary ROI analysis based on the regions that showed significant activation in the**WB** group-analysis |
| *De Zubicaray et al., 2015* | Continuous naming paradigm | SI | Perfusion signal changes as a function of ordinal position within category (cumulative interference effect) | Cluster forming threshold p<.001;  P<0.05 FWE cluster-level | left mid MTG*, left IFG/insula* and perirhinal cortex* | SVC on predefined ROIs based on a priori hypotheses |
| *De Zubicaray et al., 2017* | Blocked cyclic naming | SI | Semantically related>unrelated actions: cycles 2-6 | Cluster forming threshold p<.001;  P<0.05 FWE cluster-level | bilateral inferior frontal gyrus, left hippocampus* and left middle temporal gyrus*. | SVC on predefined ROIs based on a priori hypotheses |
| *Schnur et al., 2009* | Blocked cyclic naming | SI>PF | [semantically related > unrelated] > (phonologically related >  unrelated] | P<0.01 uncorrected | Left IFG (pars triangularis), left MTG, left middle frontal gyrus, right superior temporal gyrus, insula and lateral globus pallidus. | ROI analysis in four regions based on a priori hypotheses |
| *Hocking et al., 2010* | Postcue naming paradigm | SI | Semantically related>unrelated | Cluster-forming threshold p<0.001;  P<0.05 FWE corrected | right lingual gyrus, right precuneus, left middle temporal gyrus* | SVC on predefined ROIs based on a priori hypotheses |
| *Koester et al., 2011* | Long-lag priming paradigm | PF | primed (transparent and opaque) > unrelated | P<0.01 uncorrected | Left IFG and left middle frontal gyrus | ROI analysis in two regions based on a priori hypotheses |

*Table S5: Details of the fMRI analyses and summary of the results indicating signal suppressions. *= signal increase found at ROI level*

| **Study** | **Paradigm** | **Effect**  **(SI/PF)** | **Type of analysis** | **Statistical thresholding** | **Regions in which decreased activity was reported** | **ROI analysis** |
| --- | --- | --- | --- | --- | --- | --- |
| *Abel et al., 2009, 2012* | PWI | SI | Unrelated>categorically related | P<0.05 FDR corrected | left lingual and bilateral precentral gyri, left ACC, left posterior STG, left parietal operculum and bilateral cuneus. | In Abel et al. 2009: ROIs  selected from a predefined atlas |
|  |  | PF | Unrelated>phonologically related |  | Right inferior occipital gyrus, bilateral IFG/insula, right pre-SMA/ACC, left MTG, right STG, right brainstem, left cerebellum |  |
| *De Zubicaray et al., 2002* | PWI | PF | Unrelated>orthographically related | P<0.05 corrected (not specified) | left posterior superior/middle temporal gyrus* and right inferior temporal gyrus. | VOI correction based on a priori hypotheses |
| *De Zubicaray & McMahon, 2009* | PWI | SI |  | P<0.05 FDR corrected | LIFG pars triangularis* and orbitalis*, heschl’s*. | SVC on predefined ROIs based on a priori hypotheses |
|  |  | PF |  |  | Left and right mid-posterior and superior temporal gyri*, LIFG pars triangularis* and orbitalis*, left heshl’s gyrus* |  |
| *De Zubicaray et al. 2013* | PWI | SI | Unrelated>categorically related | Cluster forming threshold p<0.001;  P<0.05 FWE cluster-level | right parietal lobule and cuneus, left superior and bilateral occipital gyri, left thalamus, left postcentral gyrus, left calcarine, left* and right MTG, left precentral gyrus/SMA*, right SMA, left SPL | SVC on predefined ROIs based on a priori hypotheses |
| *Piai et al., 2013* | PWI | SI | Semantic incongruence vs congruence | Cluster forming threshold p<.001;  P<0.05 FWE cluster-level | left STG* | ROI analysis in four regions based on a priori hypotheses |
| *De Zubicaray et al. 2014* | Blocked cyclic naming | SI | Cumulative SI effect: cycles 2-6 | Cluster forming threshold p<.005;  P<0.05 FWE cluster-level | left MTG and ITG*, right ITG, left hippocampus* | SVC on predefined ROIs based on a priori hypotheses |
| *De Zubicaray et al., 2017* | Blocked cyclic naming | SI | Unrelated > semantically related actions: cycles 2-6 | Cluster forming threshold p<.001;  P<0.05 FWE cluster-level | bilateral occipito-temporo-parietal cortex, left inferior parietal sulcus, left anterior middle temporal gyrus* and left posterior middle/superior temporal gyri | SVC on predefined ROIs based on a priori hypotheses |

*Table S6: Details of the Regions of Interest (ROI) selected by each study.*

| **Study** | **Selected ROI** | **Method** | **ROI selection was justified by a rationale** | **A-priori defined** | **Literature reference** |
| --- | --- | --- | --- | --- | --- |
| *Abel et al., 2009* | Left AG, left mid MTG, left IFG (pars orbitalis), left STG | Predefined atlas (WFU PickAtlas) | Y | Y | Y |
| *De Zubicaray et al., 2001* | SI: STG, MTG, ACC and orbito-medial PFC | Not specified | Y | Y | Y |
| *De Zubicaray et al., 2002* | PF: left posterior S/MTG (Wernicke’s area) | Not specified | Y | Y | Y |
| *De Zubicaray and McMahon, 2009* | Left S/MTG, left IFG, bilateral ACC | 3-D probabilistic atlas (Shattuck et al., 2008) | Y | Y | Y |
| *Diaz et al., 2014* | left posterior STG, left posterior MTG, anterior STG, temporal pole, angular gyrus, pars opercularis, pars orbitalis, pars triangularis | Harvard-Oxford Cortical Structural Atlas (Desikan et al., 2006) thresholded at 25% probability level | Y | N | N |
| *Gauvin et al., 2020* | Left mid-MTG, left pMTG, left pMTG/STG, left pSTG, left IFG | 10-mm radius spheres centered on peaks in MNI space reported by previous fMRI PWI studies | Y | Y | Y |
| *Piai et al., 2013* | left superior and middle temporal cortex | AAL template (Tzourio-Mazoyer et al., 2002). | Y | Y | Y |
| *Spalek & Thompson-Schill, 2008* | Right cerebellum, left fusiform gyrus/parahippocampal gyrus, left anterior inferior frontal gyrus, left posterior inferior/middle frontal gyrus, left parietal, left superior/middle frontal gyrus | Clusters (10 or more voxels) identified in omnibus whole brain analysis (experimental tasks vs perceptual baseline task) | Y | N | N |
| *Canini et al., 2016* | Left inferior frontal gyrus, left caudate nucleus | 6mm spherical ROIs centered at activation peaks from the **WB** group-analysis | Y | N | N |
| *De Zubicaray et al., 2015* | Left mid-MTG/STG, LIFG, bilateral hippocampus, right IFG, bilateral ACC, bilateral PRc | 3D probabilistic atlases (Hammers et al., 2003; Holdstock et al., 2009) | Y | Y | Y |
| *De Zubicaray et al., 2017* | Left mid-MTG/STG, Left IFG, inferior parietal cortex, left hippocampus, motor area | 3D probabilistic atlas (Hammers et al., 2003) | Y | Y | Y |
| *De Zubicaray et al. 2013* | Left IPL, left anterior temporal cortex, left mid and posterior temporal cortex, bilateral STG, left premotor cortex | 3D probabilistic atlases  (Hammers et al., 2003; Eickhoff et al. 2005) | Y | Y | Y |
| *De Zubicaray et al., 2014* | Left mid-MTG/STG, LIFG, bilateral hippocampus, right IFG, bilateral ACC, bilateral PRc | 3D probabilistic atlases (Hammers et al., 2003; Amunts et al., 2007; Holdstock et al., 2009) | Y | Y | Y |
| *Schnur et al., 2009* | LIFG, right IFG, ACC, left STG/MTG | Not specified | Y | Y | Y |
| *Hocking et al., 2010* | Bilateral fusiform and lingual gyri, left middle and superior temporal gyri, left IFG, left SMA/medial precentral gyrus | LONI probabilistic  atlas for gray matter regions (Shattuck et al., 2008) | Y | Y | Y |
| *Koester et al., 2011* | LIFG, left posterior MTG | Predefined atlas (WFU PickAtlas) | Y | Y | Y |
